# Structural evaluation in inherited retinal diseases

**DOI:** 10.1136/bjophthalmol-2021-319228

**Published:** 2021-05-12

**Authors:** Malena Daich Varela, Burak Esener, Shaima A Hashem, Thales Antonio Cabral de Guimaraes, Michalis Georgiou, Michel Michaelides

**Affiliations:** 1 Moorfields Eye Hospital City Road Campus, London, UK; 2 UCL Institute of Ophthalmology, University College London, London, UK; 3 Department of Ophthalmology, Inonu University School of Medicine, Malatya, Turkey

**Keywords:** imaging, genetics, clinical trial, retina, dystrophy

## Abstract

Ophthalmic genetics is a field that has been rapidly evolving over the last decade, mainly due to the flourishing of translational medicine for inherited retinal diseases (IRD). In this review, we will address the different methods by which retinal structure can be objectively and accurately assessed in IRD. We review standard-of-care imaging for these patients: colour fundus photography, fundus autofluorescence imaging and optical coherence tomography (OCT), as well as higher-resolution and/or newer technologies including OCT angiography, adaptive optics imaging, fundus imaging using a range of wavelengths, magnetic resonance imaging, laser speckle flowgraphy and retinal oximetry, illustrating their utility using paradigm genotypes with on-going therapeutic efforts/trials.

## Introduction

Inherited retinal diseases (IRD) are a heterogeneous group of conditions, with unrivalled phenotypic and genotypic variability. This group includes rod-cone, cone-rod, isolated cone and macular dystrophies (MD), and cone and rod dysfunction syndromes.^1–5^ Approximately 1 in 2000 individuals worldwide are affected by this group of disorders, and 2.7 billion people are healthy carriers of at least one likely disease-causing variant linked to autosomal recessive IRD^6^; with IRD being the most common cause of blindness in the working age population in England.^7^

The huge disease burden caused by IRD^8^ and the advancements in retinal genetics, imaging and molecular biology, have led to the development of clinical trials of novel therapeutics.^9–11^ Gene supplementation, gene editing through clustered regularly interspaced short palindromic repeats (CRISPR) technology, antisense oligonucleotides, optogenetics and stem cell-based therapies are some of the techniques currently being tested to improve eyesight and/or reduce the rate of disease progression.^1–5 10 12^ The multiple on-going and upcoming trials emphasise the need for reliable and repeatable measurements, for both patient stratification and endpoint assessment. Structural evaluation is of paramount importance, since most treatments target specific cell populations and aim to halt degeneration or restore retinal structure, along with clinically meaningful functional improvement or preservation of visual function.

Multi-modal assessment of retinal architectural integrity is employed to explore disease natural history, monitor progression, inform advice on prognosis, elucidate disease pathogenesis, stratify patients and evaluate the effect of therapies. Herein, we will review the different methods for structural assessment, focusing on specific genotypic paradigms with on-going therapeutic efforts (table 1).

**Table 1:** Summary of the current and under development methods for IRD structural evaluation

Imaging modality	Characteristics	Use in inherited retinal diseases (IRD)
Colour fundus photography	Classified based on the use of confocal optics or flash-based systems. Different filters can be employed to enhance particular structures: red light → choroid; green light (red-free) → retinal vasculature, drusen, exudates; blue light → anterior retinal layers.	Fundamental tool that facilitates teaching, documentation, evaluation and monitoring.
Fundus autofluorescence imaging (FAF)	Short wavelength-FAF (SW-FAF): macula appears hypoautofluorescent. Good for evaluation of areas with photoreceptor loss but relatively intact retinal pigment epithelium, and subretinal hyper-reflective material. Near infrared-FAF (NIR-FAF): macula is hyperautofluorescent. It detects geographic atrophy and pigment migration earlier than SW-FAF.	Its property of revealing the retina’s health and metabolism makes it an important tool for diagnosing and monitoring IRD. It also provides valuable insights on disease pathophysiology.
Optical coherence tomography (OCT)	Enables highly detailed qualitative and quantitative assessments of the retinal layers.	Key tool to accurately monitor anatomical changes. Also employed intraoperatively in gene therapy clinical trials.
OCT angiography	Provides tri-dimensional visualisation of the retinal microvasculature and capillary plexi.	Useful to identify choroidal neovascularisation in association with IRD.
Adaptive optics	Two types: (i) confocal is used to resolve the cone and perifoveal rod mosaics; (ii) non-confocal (split detection) identifies cones with abnormal outer segments.	Enables non-invasive cellular imaging. Helpful to increase our understanding of IRD. Also used for monitoring progression and in research settings.
Optoretinography	Allows mapping of stimulus-evoked functional intrinsic optical signal using near infrared light.	May be useful for assessing photoreceptor integrity and dysfunction (still under development).
Laser speckle flowgraphy	Employs the laser speckle phenomenon to quantify in vivo the circulation in the optic nerve head, choroid and retina.	Has been used to correlate blood flow with other structural and functional parameters in IRD.
Retinal oximetry	Measures oxygen metabolism by capturing how haemoglobin absorbs light.	May represent an alternative way to assess outer retinal degeneration in IRD (still under development).
Functional magnetic resonance imaging	Provides high resolution imaging of the brain including the visual cortex.	Useful to assess plasticity and remodelling following visual field defects, congenital visual impairment and/or interventions.

### Colour fundus photography

Colour fundus photography (CFP) is a widely available tool to document the retinal appearance. To begin with, drawings were used for this purpose and around the end of the 19th century, fundus cameras started to become available, with constant evolution ever since, including a broad range of digital and widefield options—arguably the two most important developments. A basic feature of retinal cameras is their optical angle of view; ranging from 20° (particularly used to image the optic disc), 30° (standard retinal view), wider angles such as 45° and 60° and ultra-wide field covering 200° (approximately 80% of the retina).^13^ These devices can also be classified based on the use of confocal optics or flash-based systems. Currently, the former is most efficient, suppressing scattered light and resulting in sharp, high contrast and high chromatic images.^14^ Different filters have also been developed to enhance particular structures. Red light improves visualisation of the choroid and its pattern; green light (red-free) is best for retinal vasculature, haemorrhages, drusen, exudates and the overall retina; and blue light is used to focus on the anterior retinal layers.^15^ CFP, and particularly ultra-wide field CFP, is almost universally included as part of both standard-of-care and research visits for trials and studies in IRD, since it facilitates both documentation, evaluation and monitoring of for example, progression of areas of atrophy,^16^ treated areas and retinotomy sites, inflammatory features including vasculitis, retinitis and choroiditis,^17^ and is also valuable for teaching. CFP is frequently used for topographical tracking of functional tests, such as fundus-guided microperimetry, and moreover, can be used to overlay a wide array of functional assessments onto the retinal landscape.

### Fundus autofluorescence imaging

Fluorophores are molecules that have the capacity to emit light when excited by appropriate wavelengths, a characteristic called autofluorescence (AF).^18^ Exogenous fluorophores such as fluorescein and indocyanine green are broadly used for diagnostic purposes in ophthalmology—with their application in angiography not discussed herein given their limited utility in IRD. Endogenous fluorophores like lipofuscin can be found in most eukaryotic cells, and in the eye are predominantly in the retinal pigment epithelium (RPE).^19^ The AF signal corresponds to the concentration of lipofuscin and other secondary fluorophores, which also relates to the pace at which photoreceptor outer segments (OS) are metabolised by the RPE cells.^20^

There are two main techniques of fundus autofluorescence (FAF) imaging: (i) short wavelength AF (SW-FAF), acquired with a 488 nm blue light that excites lipofuscin and N-retinylidene-N-retinylethanolamine (A2E), with an emission range between 560 and 700 nm; and (ii) near infrared AF (NIR-FAF), where excitation occurs at 787 nm (thereby also exciting melanin located in the RPE and choroid) and emission around 800 nm.^19 21^ Both employ a confocal scanning laser ophthalmoscope (SLO) and with both the optic nerve and vessels appear dark due to lack of lipofuscin and light absorption by blood, whereas the macular appearance differs.^22^ In SW-FAF, the macular region is hypoautofluorescent, while with NIR-FAF, it is hyperautofluorescent relative to the surrounding retina.^22^ These two imaging techniques complement each other, as NIR-FAF detects geographic atrophy and pigment migration earlier than SW-FAF, while the latter is better at detecting areas with photoreceptor loss but intact RPE, and subretinal hyper-reflective material.^23^

**Figure 1:**
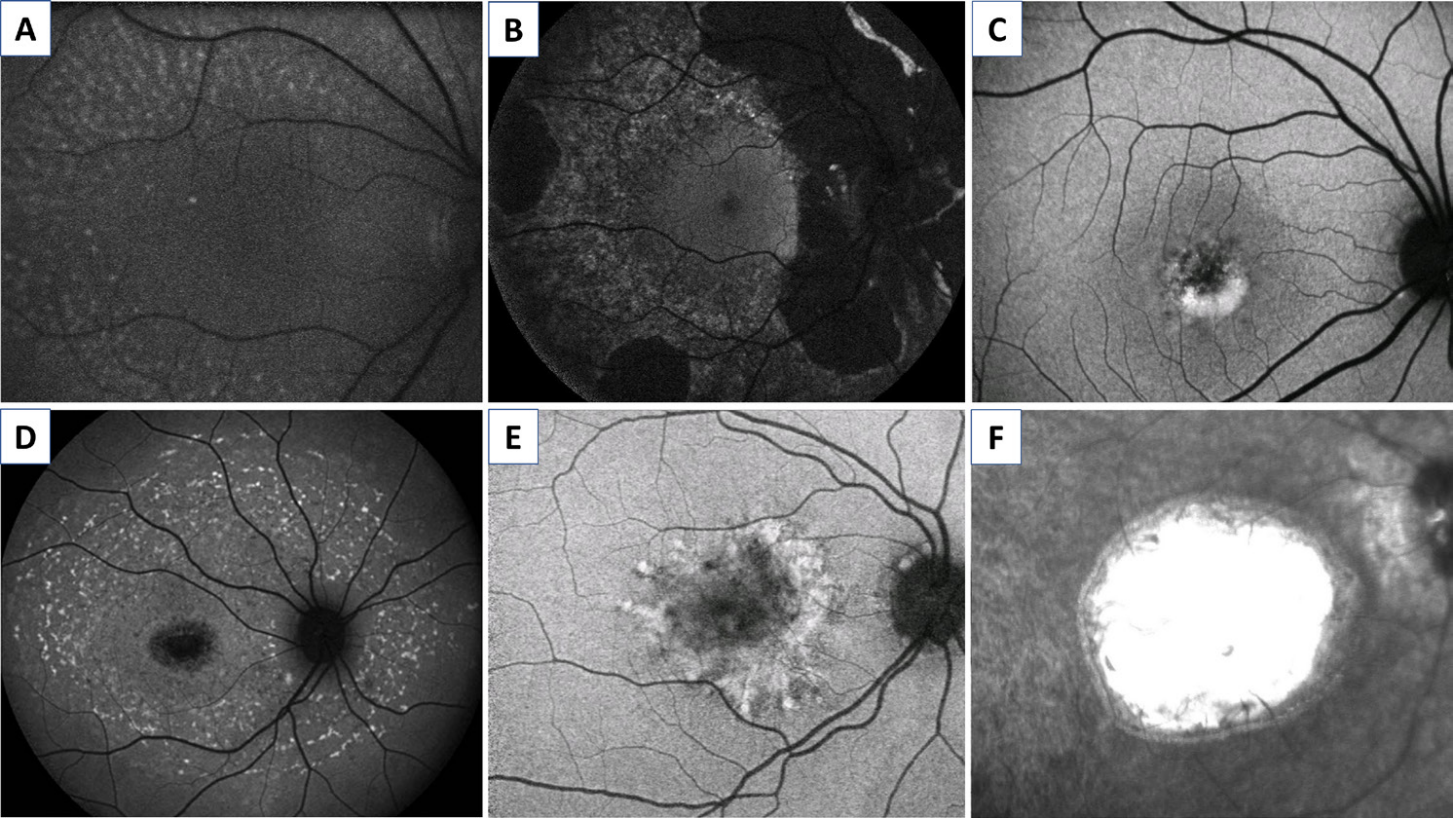
Examples of fundus autofluorescence patters in inherited retinal disease. Hypoautofluorescent defects secondary to: (A) reduced concentration of lipofuscin in *RDH5*-fundus albipunctatus, (B) retinal pigment epithelium atrophy in choroideraemia and (C) fibrotic tissue in late-stage *BEST1* vitelliform macular dystrophy. Hyperautofluorescent defects secondary to: (D) increase in lipofuscin appearing as flecks in *ABCA4*-retinopathy, (E) drusen *in EFEMP1*-autosomal dominant drusen and (F) window defect in *NMNAT1*-Leber congenital amaurosis. (A–E) Short-wavelength and (F) near infrared autofluorescence images.

Hypoautofluorescence can be due to a reduced concentration of lipofuscin (eg, *RDH5*-fundus albipunctatus, figure 1A, and *RPE65*-early onset severe retinal dystrophy (EOSRD)),^24 25^ RPE atrophy (eg, choroideraemia, figure 1B),^26^ fibrotic tissue (eg, late-stage *BEST1* macular dystrophy, figure 1C)^27^ or signal absorption by cells or extracellular material overlying the RPE (eg, subretinal bleb following gene therapy administration).^28^ Hyperautofluorescence, on the other hand, can be explained by an increase in lipofuscin (eg, flecks/vitelliform deposition in *ABCA4/BEST1/PRPH2*-associated retinopathy, figure 1D),^29^ intraretinal fluid (cystoid macular oedema),^30^ drusen (eg, *EFEMP1*-autosomal dominant drusen, figure 1E)^1^ and window defects (absence of signal blockage, eg, macular dysplasia in *NMNAT1*-Leber congenital amaurosis (LCA), figure 1F).^9 31 32^

FAF imaging has become a key tool to diagnose and monitor the progression of IRD, because of its property of revealing the retina’s health and metabolism. It has also provided valuable insights into disease pathophysiology. Generalised lack of AF in patients with EOSRD/LCA has typically been associated with variants in genes that affect the visual cycle, such as *RPE65* and *LRAT*.^33^ Nevertheless, the universal nature of this feature has been recently questioned, with retained AF shown in some patients with *RPE65*-retinopathy, mainly at the posterior pole.^25^ Hypoautofluorescent lesions have also been of interest, with their intensity varying according to the depth of the outer layer defect. Using semiautomated software, the decreased AF can be graded into definitely decreased or questionably decreased, taking as reference the hypoautofluorescence of the optic nerve head, and the rate of atrophy enlargement tracked accurately over time.^34–38^ This has been investigated in detail in multiple observational Stargardt disease (STGD) studies, and is being employed currently as an endpoint in interventional trials.^36–39^ In direct contrast, variants in genes that affect the clearance of all-*trans*-retinal from the interior of the outer segment discs, increase the accumulation of lipofuscin and related fluorophores, resulting in hyperautofluorescence. This characteristically occurs in *ABCA4-* and *PRPH2*-associated retinopathy and also underlies the perifoveal high intensity ring seen in a broad range of IRD.^1 40 41^ The latter annular pattern of increased signal has been associated with the boundary where the ellipsoid zone (EZ) starts becoming discontinuous, and also with decreased retinal sensitivity (assessed by multifocal electroretinography and fine matrix mapping).^42 43^ It is likely to represent ongoing RPE/photoreceptor stress and an intermediate stage before cell loss. The ring can be present in both cone-rod dystrophies (CORD) and rod-cone dystrophies (RCD), and by serially measuring (eg, area or greatest linear dimension) either its expansion in CORD, or constriction in RCD, disease progression can be quantified.^44^ The ring can be seen both with SW-FAF and NIR-FAF, with the caveat of being smaller in the latter. It has been postulated that this phenomenon may indicate that NIR detects earlier cellular changes, that will later become visible with SW-FAF.^45^

Qualitative assessment of the area of decreased AF is also useful for longitudinal assessment of patients with RPE atrophy.^46^ The decrease in signal has been correlated with the loss of RPE, and has been proposed as a metric in MD such as STGD, and cone dystrophies (COD)/CORD.^39 47^ In conditions such as choroideraemia and RCD, where there is conserved macular signal due to relatively preserved structure, quantification of the area of intact signal can be a meaningful measurement of disease progression or prevention of degeneration, clinically and in clinical trials, respectively.^44 48^ Ultra-wide field FAF patterns are also increasingly being used to categorise conditions such as RCD and STGD, including identifying the magnitude and extent of mid-peripheral and far-peripheral retinal involvment.^46^

A SW-SLO system using a 450 nm blue light has been used to image the retina, providing pictures that are referred to as ‘colour-FAF’.^49^ The emission spectrum can be subdivided into two images: red (560–700 nm) and green (510–560 nm). This technique provides additional information about minor fluorophores such as advanced glycation end products and oxidised fluorescent form flavin adenine dinucleotide (FAD) of the redox pair FAD-FADH2, that appear on the green, short wavelength image.^50^ Lipofuscin and A2E are mostly responsible for long wavelength emission (red image). One application for green AF has been to monitor subretinal hyper-reflective material over Bruch’s membrane,^50^ and in the characterisation of small, central lesions, given its lower absorption by macular pigments.^51^ Another tested approach is the use of SW reduced-illuminance AF, which employs a custom percentage of the laser power and can go as low as 25% of conventional intensity.^52^ Studies have shown a high correlation with standard AF in patients with STGD, but with better tolerability—bearing in mind the potential toxicity of higher intensities for patients with IRD.^53^

Beyond standard FAF, fluorescence lifetime imaging ophthalmoscopy (FLIO) is a developing modality for further functional imaging, based on the decay time of the fluorescent molecules.^54^ FLIO is a promising tool to detect and assess varying metabolic states of the retina, potentially allowing characterisation of disease areas before damage is visible with other structural imaging methods. It also enables differentiation between zones with preserved outer layers, photoreceptor loss and photoreceptor-RPE complex disruption. Hyperfluorescent FLIO rings with short FAF lifetimes may provide insight into the pathophysiological status of RCD-affected retinas, perhaps providing a more detailed/sensitive assessment of disease progression.^55^

### Optical coherence tomography

Since its introduction in 1988, optical coherence tomography (OCT) has become the most valuable tool for retinal structural assessment, providing an in vivo cross-sectional view of the retina that has revolutionised clinical and academic practice.^56^ Initially, the signals were time-encoded and these devices were referred to as time-domain OCT.^57^ Later on, spectral-domain OCT provided an improved axial resolution (from 10 to 2 µm) and faster acquisition speed, by collecting backscattering signals through a broad-bandwidth light source.^58^ More recently, swept-source OCT using rapidly tunable lasers with longer wavelength, has allowed imaging of deeper structures, improved visualisation even with media opacity, higher contrast and wider scans.^59 60^ The higher resolution of OCT not only enabled qualitative assessment of multiple retinal layer integrity, but moreover, allowed repeatable quantitative/volumetric measurements. In IRD, OCT has transformed disease characterisation, including revealing countless phenotypic features such as retinal tubulations at the margin of the outer retinal loss in choroideraemia and other advanced retinal dystrophies,^61 62^ intraretinal foveal schisis in X-linked retinoschisis,^63^ and thick, abnormally laminated retina in *CRB1*-associated disease.^64^

**Figure 2:**
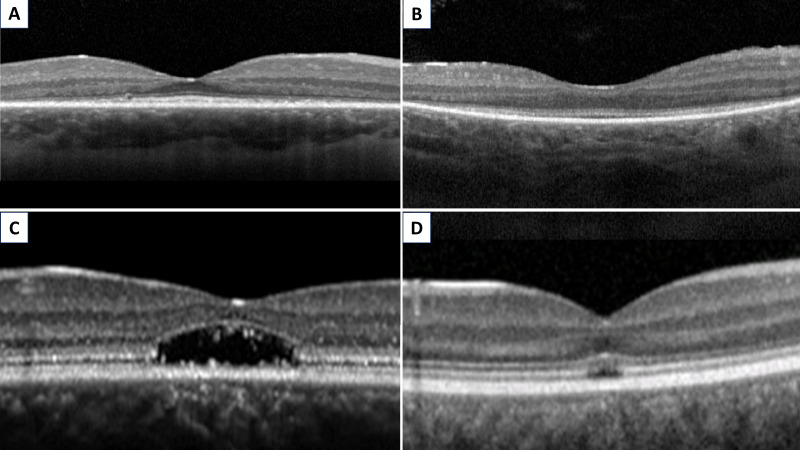
Optical coherence tomography evaluation of the ellipsoid zone (EZ). Foveal EZ preservation, with peripheral EZ loss, in (A) *RPGR*-RP and (B) *RPE65*-LCA. Foveal EZ loss, with peripheral EZ preservation, in (C) *ABCA4*-MD and (D) *CNGB3*-ACHM. RP: retinitis pigmentosa; LCA: Leber congenital amaurosis; ACHM, achromatopsia; MD, macular dystrophies.

The ability to accurately determine anatomical degeneration has changed the IRD landscape. The shortening of photoreceptor OS is one of the earliest findings in RCD and can be assessed by measuring the length between the inner surface of the hyper-reflective layer between inner segments and OS of photoreceptors, also known as the EZ, and the inner surface of the RPE.^65 66^ The shortening of this layer has been shown to significantly correlate with several functional parameters including visual field, central retinal sensitivity and best-corrected visual acuity (BCVA).^67–69^ Arguably more robust metrics include serial EZ area (EZA) and diameter (EZW, width), which have been explored extensively as measurements in RP and LCA monitoring and are being used currently as structural endpoints in clinical trials (figure 2A, B).^25 44 70 71^ In MD and COD/CORD, quantification of EZA and EZW loss can also serve as eligibility criteria and outcome measurements for intervention (figure 2C, D).^72^ EZA/EZW are metrics of great value for elucidating disease natural history in IRD. EZA loss has been shown to be greater than the area of decreased AF in STGD.^47 72 73^ These observations support the theory that photoreceptor degeneration precedes RPE loss in STGD, or that functional RPE loss precedes the structural loss of RPE leading to photoreceptor loss before structural damage becomes apparent on FAF—importantly, this is in direct contrast to prevailing pathogenesis descriptions alluding to RPE loss preceding photoreceptor degeneration,^74^ and may thereby necessitate a paradigm shift.

Other useful OCT features are volumetric analysis and thickness assessments. Parameters such as central foveal thickness, submacular choroidal thickness (measured with standard and enhanced depth imaging) and macular volume are significantly reduced in patients with RCD, when compared with normal controls.^75^ However, these latter parameters are not always correlated with visual function, thereby measuring the EZ remains one of the most sensitive ways of tracking the progression of RCD.^76^ In addition, outer nuclear layer-foveal thickness has been frequently used as a surrogate measurement of foveal cone number, even though studies employing adaptive optics cellular imaging have proved the lack of correlation between these two parameters in conditions such as achromatopsia (ACHM).^77–79^

A present challenge regarding OCT analysis in patients with IRD (particularly CORD and STGD) is that automated retinal layer segmentation tends to be unreliable when retinal architecture is altered, requiring time consuming manual correction.^80^ To solve this issue, semiautomated segmentation methods have been developed, based on the selection and manipulation of a subset of scans.^80^ These methods may provide reliable measurements within particular Early Treatment Diabetic Retinopathy Study (ETDRS) rings, resulting in relevant diagnosis and monitoring information.^81^ However, inaccuracies remain regarding segmentation of the outer retina and atrophic areas, especially in the outer ETDRS ring, which still require manual correction.^82 83^ Deep learning approaches will likely be helpful in the near future to decrease the need for costly and impractical manual correction.

OCT can also be employed intraoperatively to facilitate optimal targeting and safe treatment delivery in subretinal injections of gene therapy products in IRD.^84^

### OCT angiography

OCT angiography (OCTA) provides tri-dimensional visualisation of retinal microvasculature and capillary plexi.^85^ It has become an easier, faster and safer alternative to fluorescein and indocyanine green angiography; although not entirely replacing angiography, which can unlike OCTA, demonstrate leakage. OCTA is particularly helpful in identifying choroidal neovascularisation in association with vitelliform deposition in IRD. Multiple studies analysing the central 3 and 12 mm have shown decreased perfusion and vessel length density in the superficial and deep plexi of the choriocapillaris in patients with RCD.^86–88^ The loss of photoreceptors causes a reduction in the retina’s oxygen consumption, thereby likely leading to the aforementioned vascular changes.^89^ A significant association between these and other features, such as the width and integrity of the EZ, BCVA and visual field has been demonstrated.^86–88 90^ Recently, reduced microvascular density has been associated with a decreased number of cones, quantified with adaptive optics imaging.^91^ It has also been postulated that the observed decline of choroidal vessel density occurs at late stages of retinal degeneration and further aggravates photoreceptor dysfunction. Therefore, analysing the integrity of the choroidal vasculature may be important in predicting the progression of IRD, as well as the responsiveness to treatment.^92^

### Adaptive optics

**Figure 3:**
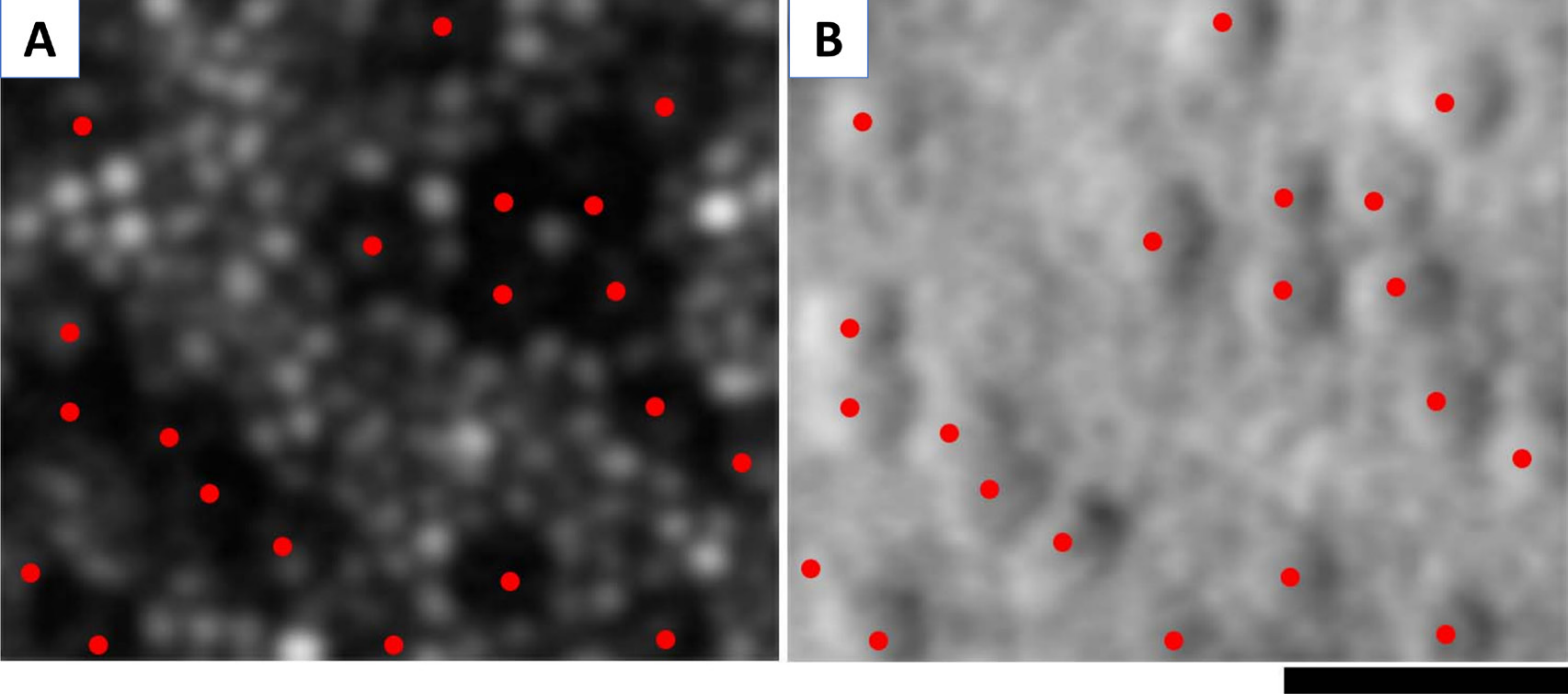
Cellular imaging with adaptive optics scanning light ophthalmoscopy (AOSLO). AOSLO imaging in *CNGA3*-associated achromatopsia: (A) confocal image, with red dots marking the ‘dark’ (non-waveguiding) cones. Cones are surrounded by waveguiding rods. (B) Non-confocal (split detection) image over the exact same region with overlying red dots showing the cones marked in (A), which colocalise with cone inner segments, surrounded by rods. Scale bar: 20 µm.

Adaptive optics scanning light ophthalmoscopy (AOSLO) allows for non-invasive cellular imaging, thereby helping to improve our understanding of IRD.^93^ An increasing number of natural history studies and ongoing/planned interventional clinical trials exploit AOSLO both for participant selection, stratification and monitoring treatment safety and efficacy.^93–97^ There are currently two main types of detection: (i) confocal, which uses the light that is backscattered by photoreceptors with relatively intact OS to resolve the cone and perifoveal rod mosaics (figure 3A); and (ii) non-confocal (split detection), that processes images capturing the light to the right and left of the confocal aperture, enabling the identification of cones with abnormal OS (figure 3B).^93^ Several metrics are often employed, including cone density and spacing, peak cone density, Voronoi analysis of the regularity of the mosaic and reflectivity.^98^

AOSLO advanced retinal phenotyping has provided novel insights into IRD. It has been described that at 1° from the fovea, a cohort of patients with CORD had 0.7 times greater spacing than a control group, and a cohort of patients with RCD, 0.2 times greater spacing.^99^ The more spacing between the cells, the less density of cones in that area. While it would be predicted that AO imaging and cone density would be abnormal in CORD, in RCD, one might expect there to be relatively preserved cone function and structure until advanced stages. However, AO imaging has shown that in patients with RCD (mean retinal sensitivity greater than 35 dB), the cone density is decreased, even in areas where the EZ appears intact on OCT. Therefore, AO can monitor patients with mild RCD before changes are evident on OCT.^100^ Moreover, by detecting change more sensitively and thereby in shorter periods of times, AO may be helpful in determining the rate of progression in a far more timely fashion compared with other modalities.^101^ Qualitatively, cone-free patches have also been demonstrated at the fovea of patients with RCD, while these were absent in the normal population.^99^ Similarly, in STGD, AOSLO has shown early parafoveal decrease of photoreceptors.^29^ Nevertheless, despite the exquisite ultra-resolution of AO cellular imaging, it is significantly limited by the lack of a commercially available compact device and the inability to acquire standardised images in the majority of patients with IRD—in direct contrast to OCT.

However, AOSLO has been successfully employed in several deep-phenotyping studies for ACHM, showing highly variable residual cone density for *CNGA3* 78 *-* and *CNGB3* 77 *-*ACHM (both genotypes have on-going gene therapy trials), better preserved mosaic in *GNAT2* 102 -ACHM and greater degeneration in *ATF6* 79- and *PDE6C* 40-ACHM. AOSLO cone counting with split-detection imaging has been reported to have good repeatably in STGD, *RPGR*-RCD and *CNGA3*- and *CNGB3*-ACHM, with variability between different diseases.^103 104^ Furthermore, AOSLO can contribute to the differentiation between entities, such as oligocone trichromacy (sparse mosaic of normal wave-guiding cones at the fovea) and bradyopsia (relatively intact photoreceptor mosaic).^105^ Nonetheless, the correlation between AO images and functional parameters is still under evaluation; with AO-guided cellular psychophysical testing still in early development—and the promise of ‘nanoperimetry’. Intriguingly, and with potential therapeutic implications, it has been reported that even with 40% of the normal cone density, BCVA and retinal sensitivity remain within normal limits.^106^

### Optoretinography

Optoretinography (ORG) is a recent technique that allows mapping of stimulus-evoked functional intrinsic optical signal (IOS) using near infrared light—‘functional imaging’.^107^ During phototransduction, a light stimulus causes slight shrinkage of photoreceptor OS by narrowing the inter-disc spacing, leading to bleaching and IOS (scattering and refractive index, among others).^108^ Measuring IOS has been correlated with photoreceptor integrity, which may, among other potential applications, have utility in stratification of patients for new therapies.^109^ ORG has been assessed in patients using high-speed OCT and AOSLO, although improved instruments and software is still required to optimise the technology.^110–112^ Preclinical studies have investigated ORG in murine models of photoreceptor dystrophy such as RCD, and shown that ORG was able to detect photoreceptor dysfunction.^113^ The perfecting of this modality may provide us with deeper evaluation of how photoreceptors function and respond to new therapies.

### Laser speckle flowgraphy

Laser speckle flowgraphy (LSF) employs the laser speckle phenomenon to quantify in vivo the circulation in the optic nerve head, choroid and retina.^114^ In RCD, LSF has been used to show that decreased macular choroidal blood flow was closely associated with reduced central visual function.^115^ These findings reinforce the need for further evaluation of choroidal blood flow in patients with IRD.^116^

### Retinal oximetry

Retinal oximetry devices have significantly advanced over the last decade, associated with improving reproducibility.^117^ Oxygen metabolism can be measured due to the different light absorption of oxy-haemoglobin and deoxy-haemoglobin. In IRD, outer retinal degeneration decreases the overall retinal oxygen requirement. Retinal loss also leads to increased diffusion of oxygen from the choroidal circulation into the inner layers, reducing the need for oxygen delivery from the retinal circulation, and ultimately causing increased venous saturation.^118^ Using retinal oximetry, oxygen saturation in retinal venules has been found to be significantly higher in patients with RCD than in controls, and the arteriovenous difference, lower.^119 120^ These findings were significantly associated with macular thickness and electrophysiology responses.^121^ Retinal oximetry’s role in IRD requires further evaluation in large genotyped cohorts.

### Neuroimaging

Magnetic resonance imaging (MRI) affords the ability to obtain high resolution structural images of the visual cortex and also sensitively record associated responses (functional MRI).^122^ Importantly, several MRI studies have described a degree of plasticity/remodelling following visual field defects in RCD or MD, and also with congenital visual impairment, including in ACHM.^123–125^ The remapping of the primary visual cortex (V1) consists of a shift of central retinal inputs to more peripheral locations in V1, and this phenomenon was found to be larger in patients with more constricted visual fields.^125^ However, it was noted that individuals with RCD did not have marked structural differences compared with controls (changes in white matter were mild); but in contrast, individuals with early-onset visual loss had thickened striate cortical and grey matter.^125–128^ It is of note that a child with ACHM who underwent gene therapy has been described who demonstrated cone-driven retinotopically organised signals in visual cortical areas, absent before the treatment.^129^ This raises the possibility of using MRI to measure ophthalmic gene therapy outcomes.

### Deep learning

Imaging in medicine is now being aided by artificial intelligence-based algorithms that are intended to reduce errors and decrease analysing time. Deep learning consists of artificial neural networks that have self-learning algorithms based on large volumes of high-quality training data.^130^ While sufficiently large databases may be challenging to obtain for all IRD, this technology is already being applied to the IRD field.

Miere *et al* have used FAF images (n=389) to automatically classify IRD into the categories of Best disease, RCD, STGD, and controls, with an overall accuracy of 95%.^131^ Arsalan *et al* chose instead CFP (n=2160) to develop a network that segments the retina and detects pigment; with an accuracy of 99.5%.^132^ Masumoto *et al* used 373 ultra-wide CFPs and FAF images to develop a platform that differentiates RCD from normal retinas, with a sensitivity and specificity of over 99%.^133^ While, Fujinami-Yokokawa *et al* used OCT scans to create an approach that differentiates between retinal dystrophies secondary to pathogenic variants in *ABCA4, RP1L1* and *EYS*, with a mean accuracy of 90.9%.^134^ Deep learning has also been used to binarize AF images from patients with RCD, and accurately identify and outline the hyperautofluorescent ring. This method showed statistically significant higher precision than subjective visual inspection.^135^ It is expected that deep learning algorithms will continue to improve and become integrated into high-definition technologies. This should help with more rapid accurate diagnosis and monitoring of disease, as well as facilitate trials, treatments and education.

## Conclusions

Advancements in multimodal retinal imaging have transformed the practice of retinal genetics over the last 10 years, and no doubt will continue to evolve and expand over the next decade. These developments have shed light on disease mechanisms, allowed more timely diagnosis (helped to shorten the ‘diagnostic odyssey’) and earlier disease detection, prioritised genetic testing, facilitated more accurate advice on prognosis and more sensitive measurement of rate of change over time. They have also helped treatment development, cohort characterisation, trial design and outcome validation. Further improvements are anticipated, including with respect to ultrastructural imaging, metabolic imaging, improved structure-function overlays/correlation, and the establishment of artificial intelligence-mediated diagnostics to improve care and opportunities for patients with IRD.
